# β-Defensins: Farming the Microbiome for Homeostasis and Health

**DOI:** 10.3389/fimmu.2018.03072

**Published:** 2019-01-25

**Authors:** Kieran G. Meade, Cliona O'Farrelly

**Affiliations:** ^1^Animal and Bioscience Research Centre, Teagasc, Grange, Ireland; ^2^School of Medicine, Trinity Biomedical Sciences Institute, Trinity College Dublin, Dublin, Ireland

**Keywords:** defensin, immune privilege, innate, microbiome, basal, constitutive

## Abstract

Diverse commensal populations are now regarded as key to physiological homeostasis and protection against disease. Although bacteria are the most abundant component of microbiomes, and the most intensively studied, the microbiome also consists of viral, fungal, archael, and protozoan communities, about which comparatively little is known. Host-defense peptides (HDPs), originally described as antimicrobial, now have renewed significance as curators of the pervasive microbial loads required to maintain homeostasis and manage microbiome diversity. Harnessing HDP biology to transition away from non-selective, antibiotic-mediated treatments for clearance of microbes is a new paradigm, particularly in veterinary medicine. One family of evolutionarily conserved HDPs, β-defensins which are produced in diverse combinations by epithelial and immune cell populations, are multifunctional cationic peptides which manage the cross-talk between host and microbes and maintain a healthy yet dynamic equilibrium across mucosal systems. They are therefore key gatekeepers to the oral, respiratory, reproductive and enteric tissues, preventing pathogen-associated inflammation and disease and maintaining physiological normality. Expansions in the number of genes encoding these natural antibiotics have been described in the genomes of some species, the functional significance of which has only recently being appreciated. β-defensin expression has been documented pre-birth and disruptions in their regulation may play a role in maladaptive neonatal immune programming, thereby contributing to subsequent disease susceptibility. Here we review recent evidence supporting a critical role for β-defensins as farmers of the pervasive and complex prokaryotic ecosystems that occupy all body surfaces and cavities. We also share some new perspectives on the role of β-defensins as sensors of homeostasis and the immune vanguard particularly at sites of immunological privilege where inflammation is attenuated.

## Introduction

Defensins are small cationic peptides, present in a wide range of species across the animal and plant kingdoms. Characterized by a conserved six cysteine signature, three sub-classes of defensins have been identified as α, β, and θ (1). α-defensins arose from β-defensins in some mammals (2) and the bridging pattern between the three pairs of intramolecular disulphide bonds made by the six cysteines structurally separates both classes (3, 4). A high number of functional α-defensins have been described in the equine genome (3, 5) which, surprisingly given the short evolutionary distance were not retained in most artiodactyls (6), including cattle (7). Similarly, only β-defensin genes have been found in birds (8), and β-defensin genes have been identified in all the vertebrate genomes so far sequenced indicating the origin of this gene family very early in mammalian evolution (9). θ-defensins are a recently acquired, primate-specific class of peptide, which are formed by the merging of α and β classes of defensins (10). However, β-defensins have been most extensively studied to date. In the mammalian lineage, multiple gene duplication events and subsequent sequence diversification has resulted in a large family of β-defensin peptides with diverse amino acid sequence and virtually identical tertiary structures based on these characteristic intramolecular disulphide bonds (11). These peptides are the most numerous among vertebrates and are the subject of this review.

β-defensins are usually translated from characteristic two exon gene structures, the first of which encodes a pre-pro-peptide while the mature peptide is encoded by the second exon, containing the six cysteine motif (12). Recent sequencing of many more vertebrate genomes has facilitated a comparative genomics approach to characterizing the β-defensin gene repertoire, and species-specific clades have been identified. Current estimates of the number of β-defensin genes vary from 14 in chicken to 29 in pigs, 38 in dog, 33 in chimp, and 48 in mice and humans (8, 13–15), although final numbers will be subject to change as more genomes are correctly annotated and copy number variation (CNV) is accurately recorded. Comparative immunological analyses have also identified specific amino acid sites under positive selection which is likely to drive additional functional divergence between species. The absence of the classic α-helical region from all bovine β-defensins suggests a divergence in the mechanisms of action which may have contributed to the expansion identified in the ruminant clade (16). The implications of the apparent loss of α-defensins from the genomes of some livestock species and the expansion of the β-defensin family in a species-specific manner are only now becoming apparent, and could potentially be harnessed to improve animal health (17).

From a functional perspective, characterization of β-defensin genes and peptides has been essentially limited to model organisms including mice, rats, and humans. The specific genomic expansions detected in non-model organism species, identified through advances in technology combined with recently improved genome assemblies, have been described but not well characterized to date. Functional characterization in non-model species may shed significant light on the selection pressures that drove the dynamic changes in gene content between species and highlight the multifunctional promise that β-defensins may hold for the development of novel methods to control infection. Here, we build a case here for an intimate relationship between the β-defensin repertoire and microfloral diversity across the mucosal surfaces of the body. Given the constant requirement for appropriate cultivation of the microbiome, and the prevention of inflammation-associated pathology, it is likely that the host defense peptide arsenal in general, and the repertoire of β-defensin peptides in particular represent useful tools with which to maintain health and reduce infectious disease burden in livestock species.

## Antimicrobial Meets Immunomodulation in Host Defense

β-defensins were traditionally viewed as exclusively antimicrobial molecules, as their induction in response to diverse bacterial, viral, parasitic and fungal infections was widely reported (18–20). As small cationic peptides, β-defensins are preferentially attracted to the negatively charged outer membranes of bacteria, with reported efficacy against Gram positive and Gram negative bacteria, fungi and enveloped viruses (21). Antimicrobial action is mediated via several mechanisms, including aggregation, pore formation, interference with cell wall synthesis, and prokaryotic membrane depolarization (22). The amphipathic nature of defensins enables them to insert into the phospholipid membrane of pathogens thereby destroying the integrity of the cell wall (10). Whereas, many *in vitro* studies have confirmed an antimicrobial role of several defensins, only a limited number of studies have verified their role in defense *in vivo*. Murine defensin 1 (DEFb1) Defb1-knock out mice showed delayed bacterial clearance from the lung (23) and increased Staphylococcal infection in the bladder (24). Expression of porcine β-defensin 1 (pBD1), an ortholog of human β-defensin 2, given at the time of challenge conferred protection against *Bordetella pertussis* in newborn piglets (25).

An interesting insight, resulting from the study of germ-free mice was the production of β-defensin precursors in the absence of infection (26). The concept of a germ-free animal was recognized more than a century ago by Louis Pasteur, although he also had the foresight to predict that bacteria-free existence is impossible (27). Generation of truly axenic mice requires that the pups remain sterile in the uterus and given what we now know about microbiotic priming *in utero* (28), these animals are not likely to be sterile.

As additional functions for β-defensins emerged (29), a broader interpretation of these molecules was adopted, leading to the term host defense peptide (HDP) (30, 31). In studies using embryonic kidney cells engineered to express various TLRs, human β-defensin-3 (hBD3) mediated activation of the transcription factor NFκB, depended on the expression of both TLR1 and TLR2 (32) demonstrating that TLR signaling is not restricted to recognition of microbial molecular patterns but also can be initiated by endogenous defensin peptides (33). β-defensins also serve to link the innate and adaptive immune responses—hBD3 can rapidly enter TLR4-stimulated macrophages and dampen the expression of pro-inflammatory genes (34). They also induce expression of the costimulatory molecules on monocytes and myeloid dendritic cells in a TLR-dependent manner by acting as chemoattractants for T-lymphocytes and immature dendritic cells (35). It seems that over the course of evolution, most β-defensins studied have acquired additional roles (a process known as neofunctionalisation) while retaining their original defense role (14). It is now becoming clear that antimicrobial and immunomodulatory functions of β-defensin peptides are not mutually exclusive and it is therefore logical not to compartmentalize their functions in either or, but to view their multifunctionality as an evolutionary work in progress, as critical elements with several roles in the complex function of defense against disease. Importantly, it is their multimodal action (Table 1) which has enabled β-defensins to retain their potency against infectious agents throughout the course of evolution (66). It is also their ubiquity of expression across mucosal surfaces that implicate them as fundamental players and sentinels of homeostasis and health.

**Table 1:** Catalog of distinct effector mechanisms documented for β-defensin host defense peptides.

**No**	**Mode of action**	**Details**	**Net effect**	**Relevance**	References^*^
1	Cell growth and tight junction formation	Cell cycle arrest and angiogenesis	Homeostatic but promotion of tumor growth also documented	Wound healing Cancer treatments	(36–41)
		Sequester lipids to prevent bacterial cell wall biosynthesis and interference with electron transport	Reduced pathogen proliferation and carriage.	Antibacterial applications	(42, 43)
		Binding to viral glycoproteins	Prevents cellular entry Reduced viral replication	Antiviral applications	(44, 45)
		Binding to fungi *- Candida albicans*	Yeast surface proteins required for anti-fungal activity	Antifungal applications	(46)
2	Direct binding	DNA uptake by host cells	hBD3 increases the cellular uptake of *E. coli* and self-DNA	ImmunoeducationVaccine applications	(47, 48)
		Binding sperm in epididymis	Reduced sperm aggregation and facilitates movement. Prevents immunorecognition in female tract by preventing binding of anti-sperm antibodies Increased sperm binding to oviductal epithelium	Treatments for fertility Potential utility as contraceptives	(49–52)
3	Pore formation, calcium and potassium channels and cell depolarization	Relevant to multiple classes of pathogens including parasites - *Trypanosoma cruzi* and *Plasmodia*	Increased permeability of mycobacterial cell envelope. HBD2 opens calcium activated potassium channels	Antimicrobial applications Calcium signaling relevant to sperm function	(53–57)
4	Induces release of cytotoxic granules, histamine and prostaglandin from host cells	Degranulation of Mast cells and enhancement of apoptosis	Pathogen destruction	Allergy Homeostasis	(58, 59)
5	Complement activation	Prevents fibrinolysis	HBD2 inhibits classical complement pathway	Anti-inflammatory applications	(60)
6	Lipopolysaccharide (LPS) binding	mBD1 blocks binding of LPS to LPS binding protein	Sequesters LPS to control action of inflammation	Anti-inflammatory applications	(61, 62)
7	Pathogen recognition receptor ligation	TLRs and NOD2/CARD15	Immune activation	Vaccine design	(32, 33, 63)
8	Regulation of gene expression	Prokaryotic cells	Inhibition of nucleic acid synthesis Reduced expression of genes involved in biofilm production	Antimicrobial including anti-biofilm for medical devices	(55)
		Eukaryotic cells	Enters macrophages to reduce - gene expression of cytokines including IL-1B and IL-17	Anti-inflammatory generally but pro-inflammatory effects have also been documented	(34, 61, 64)
9	Chemotaxis	Immature memory T cells, monocytes, DCs, Neutrophils	Immunoprofiling – particularly at mucosal surfaces	Homeostasis	(61, 65)
10	Cell maturation and T_H_1 polarization	T cells and DC cells	Maturation of cells, Immunoeducation	Vaccine design	(33, 35)

* *Reference list is not exhaustive - where multiple studies have documented the same effector mechanism, sample references have been included*.

## Farmers of the Microbiome

The numbers of microorganisms across the entire exposed mammalian mucosal surface outnumbers the eukaryotic cell number of the host by orders of magnitude, indicating that constant monitoring, and management of the microfloral diversity and flux is required to prevent systemic colonization and pathology. However, microbial infections are actually the exception in the generally harmonious coexistence of animals with immense numbers of non-pathogenic microorganisms (67). This harmony exists due to many mechanisms including:

1. Secretion of mucus from cells lining the mucosa which reduce direct contact between exogenous antigens, particularly from potential pathogens;
2. Secretion of glycosylated antibodies known as Immunoglobulin A (IgA) found in colostrum and absorbed across the neonatal gut in a brief time after birth and
3. Production of HDPs (68), including β-defensins, predominantly at epithelial surfaces. Commensal bacteria that make it across the epithelium are usually phagocytosed by macrophages within hours but some studies have shown that they can reside within dendritic cells (DCs) for several days (69).

Analysis of the microbiome complexities across tissues and between divergent species is beyond the scope of this review. Instead, we focus on evidence underpinning a role for β-defensins in modulating the host's cross-talk with commensals and how changes in their expression contribute to disease. All mammalian neonates share a common trajectory of immediate exposure to an abundance of microbes in the nutrient rich environment after birth, resulting in colonization across all mucosal surfaces. Although, age-related changes in the development of the microbiome have not been well defined in livestock species, data from humans and mice shows that the early neonatal stages are characterized by high compositional changes which ultimately settles to a core characteristic microbiome (70). Extensive inter-individual variation is also reported, which we contend would likely contribute to significant variation in phenotypic performance across traits in livestock. Whereas, the principal focus to date has been on the intestinal microbiome, commensal populations of microbes are found at all mucosal sites, as well as on the skin. It is now becoming clear that the microbiome precludes pathogens by both inter-species microbial competition and host immune stimulation (71). It is our contention that evolutionarily conserved, multifunctional β-defensins hold the balance of power in farming the microbiome, thereby regulating the host cross-talk with prokaryotes and determining the success with which their eukaryotic subjects can defend themselves against opportunistic disease-causing pathogens.

### The Embryome and Immunoeducation

For a neonatal eukaryote to emerge immunologically naive into the world would be a risky evolutionary strategy, particularly for livestock where animal densities, and bacterial loads can be high (72). Whereas, the sterile-at-birth hypothesis was accepted for years without refute, emerging evidence from humans and murine studies now indicate that microbial education of the neonatal immune system begins pre-birth via establishment of commensal microbial populations *in utero* (28). Their source, the complexities of their transmission to the uterus, and the mechanisms that regulate their proliferation within the nutrient rich environment of the neonate have yet to be established. In humans, bacteria derived from the maternal intestine have been detected in umbilical cord blood, amniotic fluid, meconium, and fetal membranes with no evidence of infection or inflammation (73). Additionally, a recent study in cattle has even postulated that these commensals could be derived from blood (74). It is proposed that this pre-natal introduction to microbial ligands (referred to as *immuno-education*) is critical for adaptive priming of the immune system and now forms the cornerstone of the Developmental Programming and Fetal Onset of Adult Disease (FOAD) hypothesis (75). This hypothesis holds that a lack of appropriate immuno-education early in childhood may result in dysregulated immune responses and the development of disease in later life. The critical implications of these findings for livestock rearing have yet to be seriously considered.

The environment during pregnancy is considered to be one of immunological privilege, where the introduction of some foreign antigens of paternal origin are tolerated without eliciting an inflammatory immune response (76). Immune privilege is not simply the absence of professional immune cells, but involves immune and non-immune cells acting synergistically to create a unique tolerogenic environment. During normal pregnancy, the fetus grows, and develops in such an environment, while, importantly, the uterus and maternal-fetal interface still retain the intrinsic capacity to respond promptly and efficiently to immunological challenge. Thought to represent an evolutionary strategy to prevent a hyper-inflammatory response immediately after birth, the immune system of the neonate exists in a state of inflammatory anergy. A pre-partum environment of immunological privilege coupled with inflammatory anergy and an immature adaptive immune system immediately post-partum requires a potent innate immune system for protection against inflammation and disease. We advocate that this need is met by the expression of multifunctional suite of HDPs, including β-defensins.

Umbilical vein endothelial cells produce *DEFB1, DEFB4*, and *TAP* (77) and additional β-defensin gene expression has been reported in bovine embryos themselves - with expression of *DEFB103B* at the 8-cell stage and *DEFB123* at the 16-cell stage (78). Originally, it was hypothesized that expression of β-defensins in the “sterile” environment of the embryo indicated that these genes may play a role in development. However, in light of the evidence supporting the FOAD hypothesis, it is plausible that embryonic expression of β-defensins may play an important role in the regulation of the maternally-derived microbiome during development. β-defensin expression has also been documented during embryo development in sheep (79), where curiously expression reached a maximum immediately before birth and did not continue to rise into the immediate post-natal period when immune challenge is likely to be highest. Although a restricted number of β-defensin genes have been discovered in the chicken lineage, *in ovo* developmental changes in expression of these genes has also been reported (80). In poultry, it is already known that some bacteria can traverse the shell (81), so there is no reason to think that some commensals cannot do the same. It is also possible that the repertoire of β-defensins induced by the maternally derived prenatal commensals differ from the suite induced postnatally by environmental microbes, adding another intriguing layer to the protective functions and potential functional complementarity of these molecules.

Whereas, low level constitutive expression is associated with the regulation of commensal bacterial growth, elevated expression may signify the presence of pathogens. The human ortholog of the bovine gene shown earlier to be expressed in the embryo (*DEFB103*), was increased in inflamed fetal membranes (82), indicating a protective role during embryonic development. Similarly, elevated hBD2 at the time of amniocentesis was positively associated with increased likelihood of preterm birth (83). Human papillomavirus has also been shown to upregulate β-defensin expression by amniotic epithelial cells (84), hinting at an integral ubiquitous protective role for these molecules during pregnancy. In addition to the prenatal microbiome, a prokaryotic “top up” occurs via inoculation from the birth canal during the birth process. Interestingly, where this does not occur, as in cesarean sections, detrimental changes to health can result (85). Although not common in livestock, cesarean section rates are increasing in cattle, some of which are associated with poorer clinical outcomes, especially under field conditions (86).

Immediately after birth, the neonate is coated with vernix caseosa, a creamy biofilm which develops on the skin of the fetus toward the end of pregnancy (87). Although not studied in livestock species, the vernix in humans includes multiple HDPs including defensins (88), which facilitates extra-uterine adaptation of skin. Unlike with humans, most mammals not only eat the fetal membranes but also extensively lick their offspring after birth which possibly spreads the protective effect of the vervix across the newly exposed neonate. Relevantly, artificially reared calves are often removed from their dam early post-partum and thereby forego these potentially protective actions but the consequences for their developing immunity has not been previously considered.

Additional microbes are obtained from the udder or from food, predominantly milk in the early post-natal period (see mammary microbiome later). β-defensin-2 (HBD2) is expressed in human breast milk and was found to be significantly higher in colostrum samples (89). The same study showed that the recombinant BD2 peptide was effective against both Salmonella and *E. coli* bacterial species. A core microbiome has also been defined in bovine colostrum, and differences in populations documented in milk between primiparous and multiparous cows (90). This finding suggests that calves born to multiparous cows may derive a different starter colonization culture which could differentially affect both the development of their intestinal microbiome, their β-defensin expression profile and potentially their subsequent disease susceptibility (91).

### Oral Microbiome

The neonatal oral cavity is the first point of contact with dietary-derived antigens and the initial colonization cultures are milk derived. In contrast to livestock species, the human oral microbiome has been defined (92). One proteomic analysis has identified over 3,700 human and 2,000 microbial proteins in human saliva samples (93). With now over 1,000 bacterial species identified thus far, the composition and activity of this ecosystem is thought to have enormous relevance to oral health and disease (94). β-defensins have been shown to be extensively expressed in the oral cavity across multiple species. In humans, HDPs are extensively produced by epithelial cells lining the oral cavity, and are referred to as guardians of the oral cavity (95). Expression of β-defensin 1 and 2 has also been documented in biopsies taken from the salivary gland in humans (96). In rats, orthologous genes (RBD-1 and −2) were localized to the acinar and striated duct cells of the major salivary glands, and expression was also shown to be responsive to bacterial endotoxin, LPS (97).

Saliva is key to the maintenance of homeostasis within the oral cavity, and cattle produce over 100 L per day (98). The fact that the majority of livestock do not develop digestive illness routinely in such a high antigen environment, especially in the context of intensive farming, shows how robust oral defense mechanisms must be. Given the studies in humans referred to above, it is likely that the salivary proteins have an enormous role to play in oral and intestinal homeostasis. A recent analysis using three different approaches identified 402 salivary proteins and 45 N-linked glycoproteins in bovine saliva, including multiple HDPs (99). In addition, as a direct result of the licking and suckling processes between mother and calf under natural rearing conditions, this extensive salivary proteome will have important implications for neonatal health as well.

A significant proportion of young calves are artificially reared on milk substitutes or on waste milk, often from mastitic cows whose milk is not suitable for human consumption. Such waste milk is known to contain high levels of pathogenic bacteria, including antibiotic-resistant *E. coli*, although studies have claimed that its use as a feed for calves do not affect health (diarrhea) or production parameters (100). However, the impact on the microbiome and on immunity in the GI tract was not examined.

### Respiratory Tract

Inhalation is a major route of disease transmission in human and livestock populations and the lung microbiome is now well-characterized both in health and disease (101, 102). The respiratory microbiome is of critical importance in livestock species as respiratory disease caused by infection is a major cause of losses, compromised animal welfare and morbidity. Pneumonia is a major respiratory disease caused by bacteria, and as a result, a particular focus has been applied to characterizing the upper respiratory tract in respiratory disease across multiple livestock species (103–105). Stress induced changes (e.g., transport) in the nasopharyngeal microbiota has also been implicated as a contributory factor to disease susceptibility (106, 107).

Tracheal antimicrobial peptide (TAP) was one of the first β-defensin HDPs characterized in bovine lung epithelial cells (108). TAP is now extensively researched for its potential role in resistance to *Mycoplasma bovis* (109), another major contributor to bovine respiratory disease. Studies using homozygous mBD-1-deficient mice showed that a loss of mBD-1 results in significantly delayed clearance of *Haemophilus influenzae* from lung, providing a direct link between β-defensin expression and pulmonary immunity (23). Investigations into human airway inflammatory disease have shown that β-defensins participate in antimicrobial defense in the respiratory tract during disease (110), and that the bronchoalveolar lavage fluid concentration of HBD-2 may be a useful marker of airway inflammation (111). One the most recalcitrant respiratory diseases affecting humans and animals is tuberculosis, caused by mycobacterial species of bacteria. Interestingly, artificial induction of β-defensin 2 (mBD2) in bronchial epithelium contributes to improved control *Mycobacterium tuberculosis* infection in mice (112).

Furthermore, the efficacy of these peptides is not limited to bacterial pathogens. In cattle, expression of multiple β-defensins has been documented in bronchoalveolar lavage from calves infected with bovine respiratory syncytial virus (113). Murine β-defensin 3 has also shown to have anti-viral effects against influenza virus, both *in vitro* and *in vivo* (44).

### Digestive Tract

The digestive tract has been the site of the most detailed microbiome analyses across all species studied to date, and a diverse repertoire of microeukaryotes have now been identified (114). However, the term “digestive tract” belies the functional complexity that constitutes a number of physiologically distinct regions—esophagus, stomach, duodenum, small, and large intestine and colon, and this is reflected in a very diverse regional-specific microbiome (70). In ruminants, additional complexity exists in the form of a multi-chambered stomach, known as the rumen. Major cellular differentiation exists in the epithelial structures across the intestinal regions between the foregut (rumen-reticulum and omasum) and hindgut (abomasum and small and large intestine). The ruminal epithelium is 4-layer stratified squamous structure, whereas the intestinal epithelium is a single layer of columnar epithelial cells protected by a double layer of mucous. Furthermore, whereas the ruminal epithelial layer lacks an underlying organized lymphoid tissue in the lamina propria, the intestinal lamina propria consists of defined Peyers patches and specialized “M” cells diffused by lymphatic follicles rich in immune cells [for review see Garcia et al. (115)].

In the newborn calf, the rumen is still inactive and rudimental. Instead, the esophageal groove routes the easily digested milk directly to the fourth stomach, the abomasum, which accounts for 70% of the total volume. Development of the rumen occurs during the first 12 weeks of life, during which time the calf transitions from a monogastric to a ruminant, essential for efficient utilization of forage based diets. It entails growth and cellular differentiation of the rumen, and results in a major shift in the pattern of nutrients being delivered to intestines, liver, and peripheral tissues of the animal (116). The physiological transition is stimulated by a defined and progressive sequence of microbial colonization and is essential to enable absorption and utilization of digestion end products from forage. As would be expected, it should be of no surprise given the functional divergence between these tissues that a region-specific microbiome has been identified in the bovine rumen (117). Mucosa-associated bacterial populations are distinct from those inhabiting ingesta, and these divergent microbial populations are associated with signature immune gene expression profiles (118). Epimural bacteria in pre-weaned calves differs significantly from content-associated community (119), and the different bacterial populations have been associated with divergent mRNA and miRNA expression profiles linked with epimural bacterial populations in the neonatal calf (120). In addition to the resident microbiome, environmentally-induced (transport and diet) perturbations in the rumen microbiome have recently been characterized (121).

Within the heterogenous environment of the intestine, β-defensins are known to keep the peace (122) by adjusting the balance among bacterial populations and to control homeostasis (123), although this has predominately been studied in humans. An important role for α-defensins has been documented in the equine intestinal tract (3), and given the intriguing differences in structure and presumably, microbial load between monogastric (horse and pig) and ruminant livestock species, may have contributed to a divergence in function of the defensin sub-classes. The absence of this class may be compensated for by the expansion of β-defensins in the rumen, although expression of β-defensins has been shown in the pig stomach, and in intestinal epithelium (124, 125). How these defensins might perform in the environment of the functional rumen remains an open question.

Short chain fatty acid (SCFAs) are a major source of energy in cattle, initially produced by the beneficial microbiota in the colon of pre-ruminant calves between 2 and 4 weeks of age, when high concentrations of lactate and butyrate are observed. These SCFAs stimulate epithelial cell proliferation leading to longer villi, tight junction, and immune system development. Interestingly, acetate, propionate and butyrate have recently been documented to upregulate BD1 and BD2 in human epithelial cells (126). Amongst the analyses of immune changes induced in response to SCFAs in cattle, a recent study showed decreases in *LAP, TAP*, and *DEFB4A* expression in rumen epithelium after infusion with butyrate (127). In sheep, maximal expression of β-defensins (*oBD1* and *oBD2*) was detected in the rumen during the first weeks of life and also in the digestive tract prenatally (128). This evidence would support a role for defensins in managing the microbial interface, especially during initial post-natal colonization of the intestine and rumen.

Comparing the epithelial transcriptome of germ-free vs. conventionally reared mice during intestinal colonization, significantly increased expression of defensins (*DEFB37* and *DEFB39*) in the tip of the colon in the latter group was reported. However, a significant reduction in expression was detected in the ileum, indicating a regional specific β-Defensin response to colonization (129). Interestingly, transgenic mice expressing human defensins protected against intestinal salmonella, again reinforcing a protective role for these molecules (130). Similarly, in chickens, dietary supplementation with butyrate led to a increased defensin gene expression in the caecum and a simultaneous reduction in *S. enteritidis* carriage (131).

The colon represents the most distal portion of the digestive tract, and the expression of HDPs has been reported in human colon tissue (132). Similarly, bovine enteric β-defensin was named after it was originally found expressed in the small intestine and the colon (133). As a result, it is proposed that their antimicrobial activity can be harnessed as a potential therapy for infectious and non-infectious diseases of the colon.

### Mammary Gland

Human breast milk has recently been shown to have a resident microbiome (134) concurrently with β-defensin expression. β-defensin 2 in human breast milk has been shown to have broad antimicrobial activity (89), which is thought to contribute to controlling the proliferation of microbes in this ideal growth medium. It is not surprising that microbial analysis of bovine milk has shown similar commensal bacterial populations and interestingly, changes in diversity have been associated with disease (135). Healthy mammary gland in cows has also been shown to produce β-defensins including TAP (136) and Lingual Antimicrobial Peptide (LAP) (137), and expression is widespread throughout the mammary gland (138). Induction of these genes have also been postulated as markers of the early response to inflammation (20) and mastitis (135).

It is likely that protection of the mammary gland is a primary function of β-defensin expression in milk. While the analysis of HDP action usually focuses on known pathogens, these peptides have not been tested for efficacy against newly characterized commensal species, so their role in regulating mammary gland microbial homeostasis remains unclear. Is has also been suggested that β-defensins in milk may help regulate the intestinal development in the neonate but this remains speculative until further functional characterization has been performed (91).

### Reproductive Tract

The female reproductive tract (FRT) is well endowed with HDPs including β-defensins (139, 140) and during each window of physiological transitions (pregnancy to non-gravid), the cross-talk between the immune and reproductive systems provided by endogenous HDPs may play important roles in dampening the immune response to foreign antigen such as sperm but also in regulating immune tolerance of an allogenic fetus during pregnancy (141). In mice, β-defensins are more highly expressed in the vagina than the uterus, with uterine levels peaking during the estrogen-dominant phase of the cycle (142, 143). The role for hormonal regulation has been supported with findings that estrogen increases *DEFB4A* expression by primary human uterine epithelial cells *in vitro* (144). In the ovine oviductal epithelium, *SBD1* expression is also increased by estrogen (145). However, estrogen appears to have an opposing effect on *DEFB4A* regulation in the human vagina, with expression in epithelial cultures decreasing when estrogen is used alone (146). A separate study indicates estrogen increases LPS-driven *DEFB4A* expression in the same model, with progesterone decreasing it (147).

Comparisons of virgin and pregnant bovine uteri showed divergent dominant bacterial phyla between groups (148), suggesting that pregnancies are established and maintained in the presence of a uterine microbiome in cattle too. For the majority of the year, livestock species are pregnant, and it is around reproduction when follicles are growing and estrogen is secreted but examination of their influence on β-defensin expression has not been examined to date. It is well established that after pregnancy the female (and specifically the cow) must shift from an immunosuppressive (or immunotolerant) state during pregnancy to a state of heightened immune activation with concurrent inflammation to expel fetal membranes, clear infection and restore homeostasis (149). Pro-inflammatory signals also regulate β-defensin expression in the FRT. Sperm and seminal plasma can activate inflammatory cytokines, and physiological inflammation in the female tract can promote beneficial pregnancy outcomes. IL-1β and TNFα drive expression of *DEFB4A* by primary human trophoblast cells (150) and *DEFB4A* and *DEFB103* in endometrial epithelial cells (151, 152). These cytokines may mediate the observed innate immune response to pathogens detected in the FRT. Bacterial vaginosis drives an increase in secretion of hBD-2, the peptide encoded by *DEFB4A* (153).

In humans, unprotected sex is also known to change the microbiome of the vagina and sexual transmission of commensal and potentially pathogenic bacteria (154). Expression of human and mouse BD1 has been documented in the lower urinary tract (155) and increased β-defensin (*HBD2* and *HBD3*) expression has also been documented with inflammation of the cervix (156), supporting an important role for peptides in mucus for defense of the FRT. It is probable that β-defensins evolved to regulate this microfloral influx during natural reproduction. Interestingly, a range of HDPs, including hBD-1 are found in the cervical mucus plug and this pregnancy-generated structure exerts antimicrobial activity against a number of relevant pathogens (157).

Bovine endometrial cells produce β-defensins (158) and these are significantly elevated during uterine disease (159). Viral infections are also thought to play a role in endometritis (160) and therefore it is of interest that β-defensins may also mediate the anti-viral response in the FRT, as hBD-2 levels in the cervico-vaginal lavage fluid of HIV-positive women correlate with anti-HIV activity (161). In contrast, gammaherpesvirus infection of the murine cervix attenuates β-defensin expression, which may explain the predisposition toward bacterial infection of the cervix observed in virally-infected animals (162).

Some additional intriguing insights have emerged regarding the role of commensal microbe populations in the human reproductive tract which have important implications for fertility in livestock species (163). For example, a commensal bacteria, *Staphylococcus epidermidis* was found in the oviductal fluid of parous mice and abolition of these populations by intraperitoneal antibiotic injection led to disturbed uterus- embryo interaction and derangement of embryo spacing (164). This new paradigm in our understanding also extends to the role of β-defensins within the male reproductive tract. Extensive expression of these molecules has been documented across the male tract of multiple mammalian species, particularly in the epididymis of male rats, mice, rams, horses and cattle (51, 165, 166) [for review see: (167)]. The epididymis is a single, convoluted duct, through which sperm progressively acquire functional competency for fertilization. Highly regionalised expression expression profiles of proteins including β-defensins give rise to a dynamic intraluminal environment (168). One particular β-defensin, (BD126) has been documented to play a number of roles critical to sperm survival, motility, and interaction with the female reproductive tract (49, 50, 169). In agreement with their broad role in reproductive physiology, a recent study has also shown that β-defensin gene knock-out male mice are infertile (170). Interference with DEFB1 function also decreases both sperm function but also with bactericidal activity (171), and interestingly adding back DEFB1 restored both functions. Investigations in livestock species are again preliminary but recently a β-defensin haplotype has been associated with sperm function and fertility in bulls (51, 172).

A remarkable recent discovery has been the identification of a seminal fluid microbiome in mice (166) and humans. Seminal fluid bacterial diversity has been linked to semen quality and HIV viral load in humans (173, 174). It is logical, given the abundance of amino acids and other nutritional substrates in basic seminal fluid, that it would promote bacterial growth and this microbiome is likely to influence the colonization of the FRT (175). In fact, researchers now propose that this microbiome impacts directly on the etiology of infertility (174). In this context, it now seems plausible to propose that β-defensins evolved to regulate the microbiome in seminal fluid and prevent the growth of bacterial populations that may be detrimental to either sperm quality or uterine health. However, detailed follow-on studies are required to further investigate this hypothesis.

## Future Perspectives—β-Defensins and Reproduction—Sperm Wastage or a Hidden Immune Agenda?

The male reproductive tract is home to the germ cells and reproductive fluids are ideal environments for microbial growth and particularly viral transmission (176). Of the millions of sperm ejaculated, very few make it successfully to the upper female reproductive tract, where fertilization occurs. Logically therefore, the majority of sperm are non-fertilizing sperm and are presumed wasted. We propose that on the contrary, it may be the case that these β-defensin-coated sperm may play a secondary role in the prevention of ascending infection through neutrophil recruitment and neutrophil mediated killing of microbes in the female tract. Recently a direct link between the microbiome and neutrophil recruitment has been established (177) and a role for β-defensins in neutrophil chemoattraction has been previously documented, albeit in a disease context (178). Further studies are urgently required.

## β-Defensins—diversity, Dysbiosis, and Disease

**Figure 1:**
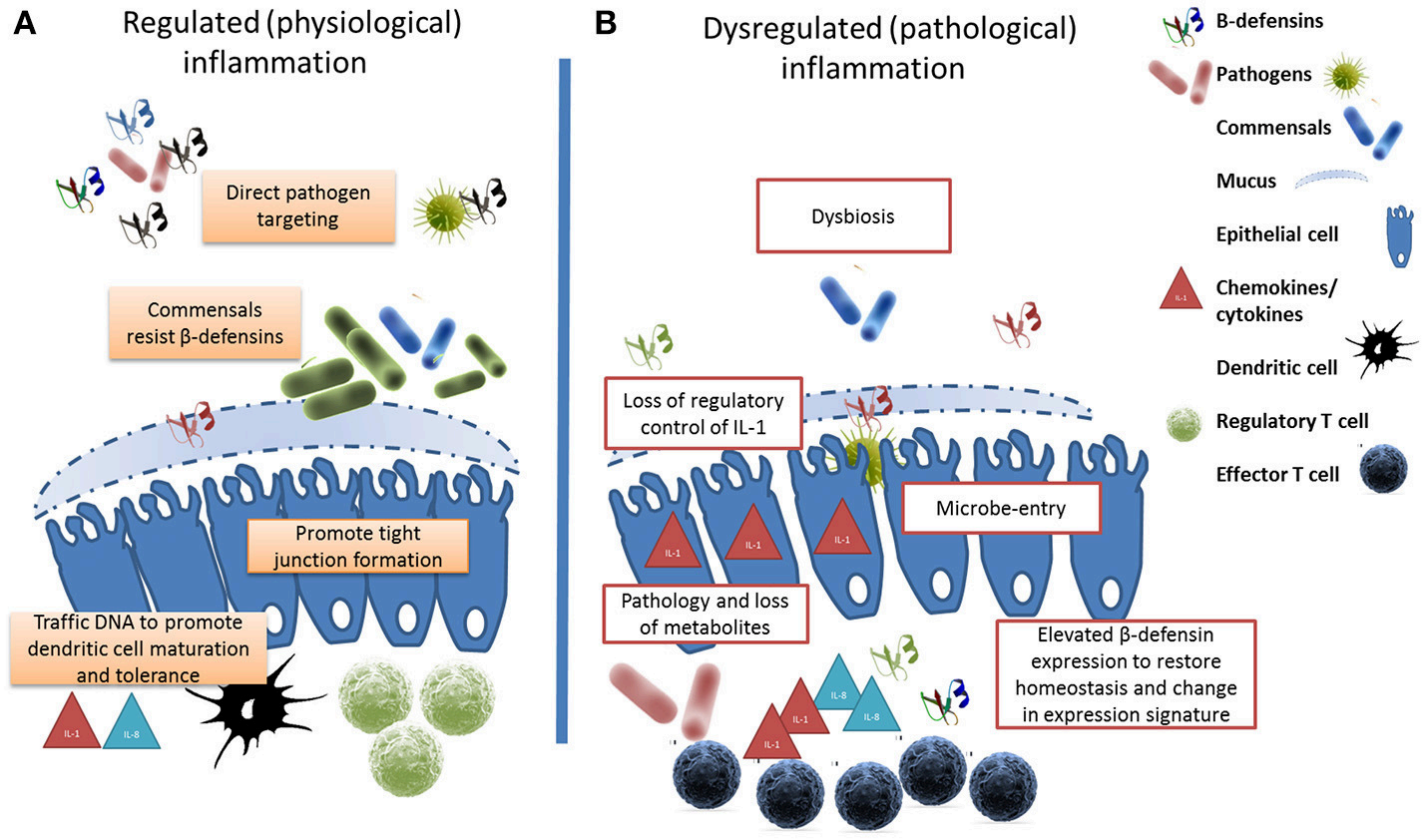
β-defensins manage the microbial interface. **(A)** Under homeostatic conditions, β-defensin-mediated preferential trafficing of microbes to dendritic cells promotes the induction of tolerance and the control of inflammation. Similarly, prenatal expression of β-defensins induces inflammatory anergy while education of the immune system occurs. **(B)** Stessor-induced dysbiosis results in dysregulation of inflammation and the loss of metabolites due to cell damage leads to a bloom of pathobionts. In an effort to restore homeostasis, elevated β-defensin expression occurs resulting in a changed expression signature. The genes encoding these host defense peptides vary in number between individuals resulting in a personal β-defensin signature which may be more or less effective at preventing a switch to pathological inflammation.

The healthy microbiome can be characterized in terms of diversity, stability, resistance and resilience (179). Compositional and functional alterations that compromise any of these parameters is referred to as dysbiosis reflected by a bloom of pathology-associated microbiota (referred to as pathobionts) and/or a loss of commensals and consequential reduction in microfloral diversity (180). Whereas, diversity is beneficial against disease (105), dysbiosis is associated with a range of pathological conditions in mice (181), humans (182), and cattle (183). The resulting dysbiosis would provide opportunities for opportunistic pathogens to invade mucosal sites, cause excessive inflammation accompanied by an associated loss of metabolites leading to dysregulated immune cell responses, pathology and disease (see Figure 1).

The current dogma suggests that colonization of mucosal surfaces begins with a period of cell-mediated immunity which is followed by the development of regulatory cell populations (e.g., regulatory T cells), which prevent excessive inflammation-associated pathology. A direct relationship has now been established between the commensal microbiome and the regulation of inflammation as the proinflammatory cytokine Interleukin 1-alpha (IL-1α) is a key regulatory target of commensal bacteria (184). Although, the mechanisms involved in immune tolerance to first colonizers of mucosal tissues are currently unknown (116), commensal (and probiotics) bacteria are now thought to contribute directly to the regulation of inflammation by the secretion of metabolites, via the prevention of LPS binding to host cells, and through the attenuation of NFKB mediated IL-1β production (185, 186). In fact, whereas a recent studies have shown commensal-induced IL-1β via the NLRP3 inflammasome (187), activation via another inflammasome complex (NLRC4) has been proposed to discriminate between pathogenic and commensal bacteria (188). Macrophage-derived IL-1α has been shown to significantly increase the expression levels of the DEFB4 in intestinal epithelium (64). This and related work in the human intestine also suggests that defensins are key regulators of bacterial diversity and thereby tissue homeostasis (122). Intestinal phagocytes are anergic to TLR ligands or commensals but constitutively express pro-IL1β, and it is now thought that HDPs initiate IL-1β posttranslational processing (189). The close association between a dysregulated microbiome and altered β-defensin expression has been most intensively studied in the context of the chronic inflammatory diseases of the human intestine known as Inflammatory Bowel Disease (IBD) (190). Clinical studies have linked the defective expression of β-defensins to the reduced killing of certain microorganisms by the intestinal mucosa of patients and directly couple dysbiosis to primary β-defensin immunodeficiency (182, 191).

The epithelium in inflamed intestinal segments of patients with Crohn's disease is characterized by a change in tight junction protein content and composition (192), resulting in barrier defects leading to luminal antigen uptake which causes mucosal inflammation. The tight junction is dynamic, multi-protein complex that forms a selective permeable seal between adjacent epithelial cells and demarcates the boundary between apical and basolateral membrane domains. Apical tight junction proteins are critical in the maintenance of epithelial barrier function and control of paracellular permeability to prevent disease (193) and an established virulence mechanism for pathogens is to limit pathogens by stabilizing tight junctions (194). HDPs, and specifically defensins are now thought to promote the resolution of inflammation and endotoxin resolution via the formation and maintenance of tight junctions (195). Defensins have also been proposed as potential markers of mucosal permeability (196). HBD-3 increased the expression of several claudins, elevated the transepithelial electrical resistance, and reduced the paracellular permeability of keratinocyte layers (36).

Although it seems the consensus from most studies is that these peptides are immunosuppressive (197) and attentuate inflammation (198), there are reports that some β-defensins amplify the immune response [as reviewed by Semple and Dorin (199)]. Some studies report a positive correlation between HBD2 expression levels and that of the potent chemokine IL-8, leading to additional inflammatory cell recruitment and acceleration of the pathogenesis of IBD (200). HBD2 has also been shown to activate pro-inflammatory cytokine expression (201) in peripheral blood cells, with similar reports for HBD3 (32) in macrophages. However, it is difficult to effectively compare between studies with various disease models, as the induction of β-defensins may be a protective attempt by the host to reduce exacerbation of the inflammatory cascade and will likely be affected by the stage of disease, the epigenetic landscape and microbial load. Furthermore, studies have also demonstrated that the structure of the β-defensin peptide will determine the biological effects detected—for example, the canonical structure of hBD3 is required for its to enter macrophages and exert its immunosuppressive effects (34).

## β-Defensin Immunogenetics

**Figure 2:**
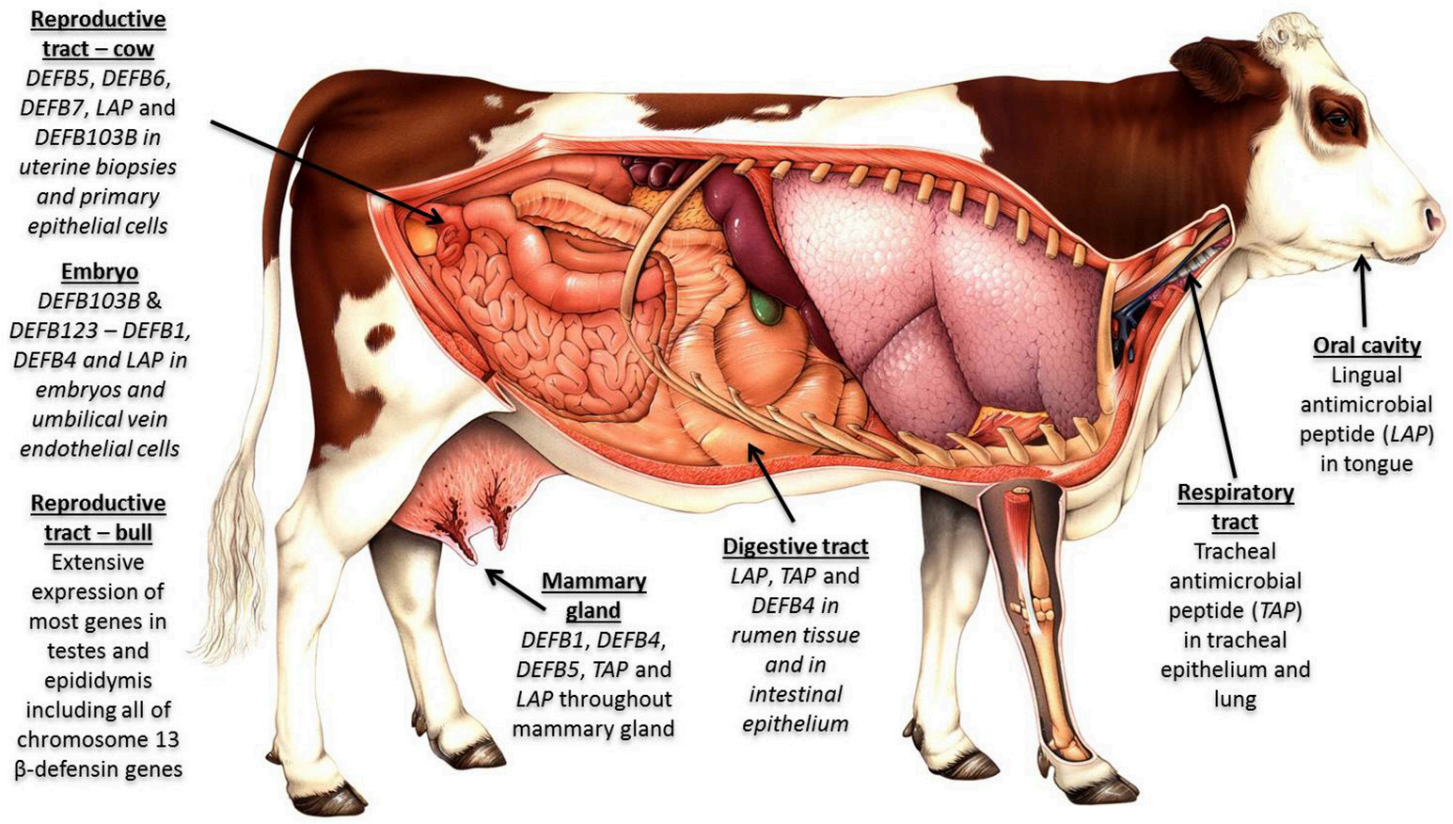
β-defensins as regulators of the microbiome and biosensors of immune homeostasis: Expression of β-defensin genes in bovine tissues—four genomic clusters of these genes exist in cattle on chromosome 8 (4 genes); chromosome 13 (19 genes); chromosome 23 (5 genes); and chromosome 27 (30 genes). The β-defensin genes on chromosome 27 are the least well conserved between species, are known to be CNV and show an expansion in number in cattle. Tissues with immunological privilege (reproductive tract) show particularly extensive β-defensin expression patterns.

Host genetic variation has a significant effect on the microbiome across multiple body sites (202, 203), and we contend that the extensive variation uncovered in β-defensin genes contributes to phenotypic diversity in several livestock-relevant traits. Recent studies have associated individual SNPs in β-defensin genes with health and production phenotypes including somatic cell count (204) and milk constituents (205). More recently, in cattle, haplotypes have been uncovered which regulate important traits like bull fertility (172). The widespread expression profile for these genes in cattle is shown in Figure 2.

β-defensins evolved through a complex mechanism of duplication which has led to highly polymorphic gene CNV (206). β-defensin gene CNV can affect disease resistance (207, 208)–it is hypothesized that increased gene-copy number contributes to susceptibility to inflammatory or autoimmune diseases but is protective against infectious disease (209). Although data in cattle are limited, β-defensin loci account for 7 of the top 25 most CNV regions across the bovine genome (210, 211). Upwards of 20 gene copies are seen for some immune genes in various cattle breeds sequenced, including for LAP, TAP, and DEFB5, genes which have been previously shown to be upregulated in response to infection, particularly in mammary, lung and uterine tissues [for review see Meade et al. (17)].

Several multigene families are subject to birth-and-death evolution and rates of gene gain and gene loss vary considerably between closely related species or even between individuals of the same species (212–215). These genes represent a major source of new (or additional) biological functions through a process known as neofunctionalisation (216). Selection for noncoding regulatory regions in hBD103 in human populations from Asia has also recently been described (217), which is thought to be a response to selection pressure from influenza viruses in the region. In cattle, the selection pressure thought to contribute to the β-defensin expansion was originally the evolution of the rumen (218). Rumen specific defense mechanisms are important to ensure the balance between immune surveillance of the diverse gut microbiota (219) and the maintenance of the integrity of the gastrointestinal epithelial barrier (123). However, the presence of these genes in monogastrics including horses (220) and pigs (221) supports an alternative rationale. Furthermore, biological systems most affected by changes in the number and organization of genes in the cattle lineage during evolution include reproduction and immunity as well as lactation and digestion (218). The intensive artificial selection that cattle have been selected to over the last 60 years, is likely to have further shaped the β-defensin genetic variation remaining in extant breeds.

The extensive expression of the expanded repertoire of β-defensins across the reproductive tract of mammalian species highlights the importance of protecting the gametes over the course of evolution. The herd breeding structure of most mammalian species has been postulated as a potential selection pressure where an expanded β-defensin peptide family would provide enhanced protection against ascending infections of the reproductive tract. Intriguingly, the genomes of avian species have not undergone expansion of these genes, but the loss of the penis in males of most avian species (222) may preclude the requirement for an expanded β-defensins family. Male ducks and Ostrich members of this clade have retained their penis and although annotation is far from complete, the duck genome is currently estimated to contain 16 defensins distributed over 3 scaffolds, a number that was slightly higher than that of the 14 defensins found in chicken (223). A preliminary search of available Ostrich genome sequence identified a number of conserved 6 cysteine sequences in addition to the currently identified β-defensin family members (unpublished data) potentially representing a β-defensin expansion. However, conclusive evidence is not yet available and it is likely that other selective pressures account for the expansion detected in the mammalian lineage.

## β-Defensins—Decoupling Inflammation From Defense

A more convincing selection pressure contributing to the expansion of the β-defensin repertoire in mammals is their requirement to maintain homeostasis in environments with a high microbe diversity as well as immunoprotection at sites of immune privilege. Inflammation in specific body sites (e.g., brain, eyes, testes) would be detrimental to the survival of the species causing pathology and possibly death. In immunopriviledged sites, immune molecules (including IL-1) can be present at high concentrations without causing inflammation indicating important immunoregulatory mechanisms. Immune cell phenotype, including regulatory T cells and macrophages, as well as the expression of anti-inflammatory cytokines is known to be critical to maintaining a functional state of hypoactivity (224). Therefore, defense against pathogens is orchestrated by alternative means, and β-defensins are likely to have a principal role.

The testes are one such site (225), where haploid gametes require protection and isolation from inflammatory immune cells and in this environment, regular inflammatory mechanisms are attenuated. The blood-testis barrier is maintained by several types of cell junctions, which limit the access of systemic immune contents to the inner reproductive compartments (225). Cytokines and chemokines are expressed in healthy tissue and function during normal testicular development (226), and increased concentrations of inflammatory cytokines have been associated with increased reactive oxygen species and histone abnormalities in sperm (227). Whereas, levels of the anti-inflammatory cytokine IL-10 in bull seminal fluid is positively associated with sperm motility, inverse correlations was detected with proinflammatory cytokines (228). High levels of the chemokine IL8 has also been positively associated with human sperm defects (229). It is our contention that the requirement for protection of the gametes and reproductive tract over the course of evolution led to the expansion of β-defensins in this regulated environment, and this thesis is supported by the extensive expression of these molecules in the epididymis across all species in which they have been studied to date (230). The requirement for β-defensins in immunopriviledged sites is not limited to the male reproductive tract and may also explain their documented expression in female reproductive tissues during pregnancy and also in the brain and eye (231).

Interestingly, studies on LPS stimulated macrophages shows that TLR-induced genes fall into two distinct categories on the basis of their functions and regulatory requirements. Referred to as “proinflammatory” and “antimicrobial genes,” a tailored innate immune response is controlled via epigenetic modifications to individual promoters (232). Methylation and chromatin modifications thereby permit the decoupling of proinflammatory from antimicrobial effector mechanisms of the innate immune system which maintains protection of the host whilst simultaneously minimizing inflammation-associated pathological damage. Such mechanisms are now known to form the cornerstone of the developing paradigm of innate memory, and have enormous relevance for the understanding of LPS tolerance, immune cell anergy and immunosuppression.

Evidence for epigenetic regulation of β-defensin genes supports this concept. The epigenetic enzyme, HDAC1 has been shown to controls BD in lung epithelial cells (233). Further work identified that DEFB1 expression was associated with specific histone marks (234). Interestingly, ablation of the microbiota has genome-wide epigenetic effects (235), and can affect transcription factor binding (236) thereby providing a direct mechanism by which commensal bacteria can regulate the immune response in a gene-specific manner. However, epigenetic changes at HDP gene promoters have not been hitherto examined.

## β-Defensins and the Response to Vaccination

β-defensins may also prove useful directly as broad-spectrum adjuvants which are required for improved vaccine design in cattle (237). As an endogenous ligand for Toll-like receptor 4 (TLR-4), inducing up-regulation of costimulatory molecules (33), β-defensin expression is thought to contribute to the establishment of a beneficial Th1 response via dendritic cell activation and increased expression of cytokines such as IFNy, IL-12 and IL-6 (238). It has been suggested that β-defensins form antigen complexes (in a manner similar to how they bind to sperm) which may be an important mechanisms of microbe trafficking to antigen presenting cells [as reviewed by (239)] which would enable appropriate education of the neonatal immune system as well as their priming during the formation of innate and adaptive immune memory. *In vivo* murine models have shown that β-defensin 2 promotes anti-tumor NK and beneficial T cell responses (240).

β-defensins are known to share significant structural similarity with chemokines and a major known mechanism by which defensins work is as chemoattractants for immune cells. Mediated via both CCR2 and CCR6 receptors (35, 241), β-defensins thereby attract myeloid and lymphoid cells to mucosal sites where they are expressed, and thereby linking innate, and adaptive immunity. They also function to increase the uptake of DNA, and promote Interferon α expression (242) which further suggests their utility in enhancing vaccine responses. DCs are usually found in mucosal tissues where they monitor the local environment for signs of pathogenic (or commensal) invasion and are integral to ensuring that pathological immune responses to harmless antigens do not develop (243). It was commonly considered that these commensal containing DCs were confined to the respective mucosal immune systems, where they prime tolerant responses of B and T cells (244). However, more recent studies have shown that homing of these DCs to the lymph nodes can lead to the development of regulatory cell populations which promote systemic tolerance (245).

Defensins are also found in B cells (246, 247), confirming that these peptides are capable of contributing to a prolonged cellular and humoral response to a pathogen (248), and this has potentially important consequences for vaccine development across all species. Therefore β-defensins are the recruitment portal through which systemic immunoregulatory DCs are recruited and thereby directs the priming of the adaptive immune response (249). This implies that neonatal β-defensin expression profiles, in regulating microbiome development, can regulate vaccine adjuvancy and thereby the efficacy and duration of protection provided (245). These studies have critical relevance for the design of next generation vaccines and adjuvants in livestock species.

## Commensal Mechanisms to Resist HDP Antimicrobial Action

A key focus of future research will identify the multiple mechanisms by which commensals can tolerate or resist the antimicrobial effects β-defensin HDPs. Large structural variations in LPS antigens have been documented across bacterial phyla, leading to different host efficacy of LPS sensing and resulting immunogenicity (250). Predominant commensal members of the human gut microbiota show temporal stability despite exposure to the host inflammatory response, but the mechanisms involved are poorly understood. A recent study identified that the dominant gut phyla in the human intestine are resistant to HDPs, and that the resistance mechanism of the common gut commensal *Bacteroides thetaiotaomicron*, is via an LPS modification (251). The authors found that the bacterial phosphatase LpxF catalyzes the removal of a phosphate group from LPS, thereby reducing AMP-dependent membrane disruption. As LpxF orthologs are widespread, this strategy may be common across gut commensals to resist host inflammation.

Furthermore, the absence of virulence factors in commensal bacteria within the bovine rumen has been suggested as key to adaptive processes that result in their exploitation of epithelial tissue for nutritional benefit as well as subversion of a detrimental immune response (252). However, full characterization of the diverse bacterial strains which exist within the microbiome will take some time.

## Concluding Remarks

Re-evaluation of the interrelationships between the microbial world and their eukaryotic subjects is well underway. It is now clear that physiological sterility is essentially impossible and microbiomes are being documented across sites from the developing fetus to healthy breast milk, tears and semen. Although these findings could have major implications for the design of new therapeutics, care is warranted and careful validation is required (253) before translation of these findings from a limited number of studies to other species can be advocated. However, untargeted, black box approaches to disease prevention or treatment are no longer seen as likely to provide long term protection. In contrast, a new appreciation for our resident prokaryotic kingdom is emerging and it is increasingly seen as key to homeostasis and health. Although bacteria are the most abundant component of the microbiome, and the most intensively studied, the microbiome actually also consists of viral, fungal, archael and protozoan communities (184, 254–256), about which comparatively little is known (184, 257–259), even in model species.

β-defensins may even have a neurological role (260). The ability of the microbiome to regulate serotonin production identifies mechanisms by which they might also regulate neural chemistry (254). Through their inhibition of glucocorticoid elevation in response to stress, defensins are even thought to prevent stress-induced immunosuppression (255). Although at first glance, the psychological effects of the microbiome in livestock species may not seem relevant, stress is of increasing concern in ethical animal production systems. Stress is a critical causal factor that not only contributes to lost production and risks to human welfare, it can also affect disease susceptibility. One study in cattle found that dexamethasone-treated calves had significantly lower expression of TAP and LAP β-defensin expression in the lungs, which was claimed as a critical contributing factor to disease susceptibility (256). β-defensin expression has been documented in human cerumen of the ear (261) and the recent discovery of β-defensin expression in the pituitary gland of fish (262) and in the murine brain (263, 264), suggests that defining the full range of β-defensin activities have yet to be defined.

Consequentially, in the absence of sterility, basal (or constitutive) expression *in vivo* may not exist. Perhaps, basal expression, of β-defensins for example, tells us a lot more than we realized previously and may provide insights into the status of the microbiome which will inevitably affect every phenotype of interest and ultimately disease outcomes. High basal expression may actually represent an induced response indicative of colonization (*immunoeducation*), infection or indeed identify eukaryotes with a more robust prokaryotic management system, and therefore an immune advantage. Furthermore, the occurrence of mysterious “natural antibodies” at birth may reflect priming that occurs during *in utero* immune programming.

The use of antibiotics has been shown to enhance intestinal colonization of enteric pathogens (71), and have enormous significance for animal production systems. New green antimicrobial drugs are urgently required in medicine and in veterinary medicine in particular (265). Although significant limitations, both technological and economic hamper the current therapeutic use of β-defensins, these are not insurmountable and still offer hope for the rationale design of new effective drugs (266). HDPs have been proposed as potentially useful alternatives to antibiotics in feed (267), and they exhibit significant potential to improve intestinal barrier function, animal health and productivity (268). A recent study showed that feeding them to juvenile ruminants led to increased body weight, average daily weight gain, enzymatic activity and positively influenced on ruminal fermentation (269). In addition, goats treated with AMPs had higher rumen microorganism diversity indices than the control groups. For new treatments to be successful, selectivity is required—and all available evidence to date suggests that HDPs are ideally suited to this role.

β-defensins have important roles not just for the prevention of infectious disease, metabolic diseases are a major limiting factor to livestock production systems (270), and are set to grow in importance as agricultural production intensifies, and are regarded as perturbations associated with production. An exquisite symbiotic relationship therefore exists between the microbiome and the metabolome where metabolites secreted by both commensal bacteria and their host are key to maintaining homeostasis (271). As regulators of the microbiome and thereby the cellular metabolomic milieu, β-defensins have a key role in this regard (272). Nutrients (like SCFAs) directly regulate systemic energy homeostasis is key to productive and healthy livestock for human food production (273). They also directly influence immune cell development, as butyrate was previously shown to induce a murine Th1 response (274).

The relevance to agriculture is not limited to improving livestock health, but are also critically important to reproduction. Interference with DEFB1 function has been shown to decrease human sperm function and bactericidal activity (171), and interestingly adding back DEFB1 restored both functions. Deletion of a number of murine β-defensin genes resulted in abnormal sperm structure, function and sterility in male mice (275). Similar work in cattle identified a β-defensin haplotype has been associated with sperm function and fertility in bulls (51, 172) making them molecules of major interest to agriculture.

In conclusion, any definition of a commensal or pathogen is problematic given what we now know about microbiomes (276, 277). To view the ecosystem of a microbiome as a ‘battle of *good* vs. *bad* bugs' is simplistic in the extreme. We contend that regulating the composition and diversity of commensal populations is just as important a feature of HDP function as preventing invasion by pathogens. We therefore view β-defensins as sensors of the microbial equilibrium and guardians of homeostasis. It is possible that the neonatal β-defensin gene or protein signatures may be diagnostic for homeostasis across mucosal sites and prognostic for dysregulated immunity and disease later in life. What is clear is that colonization by a diverse microbiome occurs across eukaryotic surfaces, both internal and external, begins pre-birth, and incessant quantitative and qualitative pruning of the microbiome is critical to health and homeostasis (278). The cumulative evidence presented here provides strong support that without active cultivation of the microbiome by β-defensin HDPs, pathology and disease would be inevitable. We predict that stimulating β-defensin expression will emerge as a key therapeutic mechanism to enhance natural immunity, restore homeostasis and reduce the burden of infectious disease in livestock.

## Author Contributions

All authors listed have made a substantial, direct and intellectual contribution to the work, and approved it for publication.

### Conflict of Interest Statement

The authors declare that the research was conducted in the absence of any commercial or financial relationships that could be construed as a potential conflict of interest.
